# Dental Jottings

**Published:** 1871-11

**Authors:** H. L. Sage


### DENTAL REGISTER.

Vol. XXV.] NOVEMBER, 1871. [No. 11.

### Communications.

### DENTAL JOTTINGS.

BY H. L. SAGE.

*Roots of Teeth and the Maxillary Sinus.*

It is well known that the roots of the superior bicuspids and molars, sometimes pierce the floor of the antrum, while at other times, there is a mere septum of bone separating the apex of the root of a tooth from that cavity. Again there *are times* when circumstances require the extraction of these teeth—and it is not a rare case, if the attempt to do so, does not at the first endeavor, prove successful. The tooth may be broken off, leaving the root or roots, or only a portion of one may be left in the alveolus.

In attempting to remove the remaining fragment, there may be danger, when the above conditions present, if great care is not exercised, of forcing it into the maxillary sinus. In endeavoring to extract the apex of a root with the elevator, by inserting the point between it and the alveolus, this accident may happen.

Harris cites a case reported by Fauchard, "in which a canine tooth had been driven into it," (the antrum) though in a different manner from that above stated.

He speaks of it as "the only case on record," that "it was followed by severe pain, and ultimately gave rise to a tumor upon the cheek, near the nose, and three fistulous openings," which only disappeared on the removal of the canine from the cavity. The following, is a report of a case, in which the consequences were happily, not serious.

In May, 1865, Mr. G. P., aged 35 to 40, called to have the second left superior molar extracted. In the attempt to remove it with the forceps, the anterior buccal root was broken off near the apex. In endeavoring to remove the fragment with the elevator, it was suddenly forced into the antrum, though very moderate force was employed. After *fishing* for it quite a long time unsuccessfully, the attempt was abandoned. Being somewhat timid and inexperienced, the opening was not enlarged, as a bolder operator might have suggested, but in accordance with the advice of an older practitioner, the result was watched, pending further steps in case of the appearance of any trouble therefrom. But none has been produced up to this time. The floor of the antrum at that point, was about the thickness of ordinary writing paper. Length of broken end of root, about three-eighths of an inch. It would not be surprising, if such accidents were of more frequent occurrence than comes to our knowledge. Though the foregoing case may afford an example of erroneous practice, inasmuch as the root was not removed from the cavity, it is cited as showing the danger that may exist, even in so simple an operation as the extraction of the root of a tooth. At another time, and with another individual, the presence of a foreign body in the maxillary sinus, might be productive of unpleasant consequences.

*Treatment of the Maxillary Sinus, in connection with Alveolar Abscess.*

In connection with the treatment of the right superior second bicuspid, and the adjoining first molar, the following conditions presented: May 6, 1870, Mrs. G. A. G., of Scotch extraction, age about 40, temperament sanguine, called. History: in 1867, examination was made of the second bicuspid, and finding it dead, the pulp and crown cavity was cleaned out and a temporary test filling inserted; when sufficient time having elapsed and no evidence of disease having been manifested, it was on the 14th of July permanently filled with gold.

According to the statement of the patient, the tooth was filled about five years previously, and was alive, but trouble being soon after experienced, it ceased, upon the lancing of the gum. On the 6th of May, 1870, she appeared with the gum and cheek badly swollen, and the eye partially closed. On lancing the gum, which had no fistula, nearly a tablespoonful of pus was discharged. The alveolar plate was perforated, and there was much loss of bone.

Injected creosote through the perforation, leaving cotton saturated with iodine in the orifice, and discharged the patient for a few days.

May 12. Swelling much reduced and no discharge *through the gum.* Dismissed for trial.

July 13. No appearance until this date. Some swelling and a permanent fistula. No pus oozed from it. Enlarged the opening in the alveolar plate for the purpose of smoothing off the rough borders, and if necessary, the apex of the root, and continued the former treatment when, July 14th, in syringing out the cavity produced by the abscess, much force being used, a dark colored, bloody fluid issued from the nostril, from the natural opening of the maxillary sinus—indicating that the contents of the abscess discharged into, or had connection with this cavity, and which was also apparent on the insertion of the point of the syringe.

On the presentation of this state of the case, it was deemed best to extract the tooth, which was done. Though the patient had complained of being troubled with the catarrh, there had been, aside from that, no uneasiness until recently, and the discharge into the maxillary sinus, may have been of short standing. It was syringed with tepid water and a sol. of chlo. of zinc—5 grs. to the ounce of water.

July 16. Face somewhat swollen. Syringed with a mild solution composed of tinct. iodine, carbolic acid, glycerine and water.

Natural opening of the antrum apparently closed by inflammation. Left cotton in the orifice of the opening leading from the socket of the bicuspid, with direction to renew it daily, and masticate on the other side.

Dismissed, intending no further treatment except it became necessary.

August 8. The opening where the bicuspid was extracted was not closed. There was a swelling over the adjoining right superior first molar, which, on pressure, in the direction of the opening, caused an exudation of pus from the latter, so that there had been a discharge from above the molar, through a channel leading to the cavity in the bone above the bicuspid. As the abscess over the second bicuspid root extended to, or was connected with the antrum, endangering its integrity, it was not thought best to run any risk of further irritation by the treatment of the molar—consequently it was also extracted, when it presented the appearance of having been diseased for a long time and was very offensive. On removing a well inserted filling in the posterior proximal surface, it revealed the fact that either the pulp at the time of filling was nearly, or quite exposed, or was already dead. It had two roots only, of which the buccal pointed anteriorly, and was separated from the maxillary sinus by a thin septum of bone, while the lingual root pierced it, or else the portion of the floor at that point had been destroyed.

Neither of these teeth, when treatment was first commenced, had fistulous opening in the gums, though badly abscessed, and it would therefore seem that the pus escaped to the antrum through the passage made by the destruction of the floor—though the dull, heavy, uncomfortable feeling was not present at any stage of the disease—at least to any great extent.

No further treatment was resorted to, and the case was not again seen until September 7, 1871, the patient during he interim having suffered from an attack of remittent fever.

No further trouble had been experienced in the region of he maxillary sinus, while it and the parts adjacent seemed o be in a perfectly normal condition.

### *Singular Location of Dental Caries.*

Mrs. M., the wife of a clergyman, called July 22, 1871, for advice with reference to the left superior central incisor, which had been abscessed for eight years, but within four weeks the parts around had assumed an acute form of inflammation, rendering them very sensitive, and the tooth exceedingly loose. An examination failed to reveal the cause, as the tooth was, apparently, perfectly free from caries and very dense in structure, neither could any cause of death be ascertained from the history of the case. At a subsequent examination, after some treatment, a very careful and thorough exploration was made with an excavator, and a cavity discovered on the lingual portion of the root, under the gum, about midway of the distance from the neck of the tooth to the apex of the root. It was completely hidden from view, and the decay had progressed to such an extent, that the root was very nearly severed. May not the location of a cavity three lines or more above the margin of the gum, on the lingual surface of the root of a central incisor, be cited as a rare case?

